# Identifying target organ location of Radix Achyranthis Bidentatae: a bioinformatics approach on active compounds and genes

**DOI:** 10.3389/fphar.2023.1187896

**Published:** 2023-08-10

**Authors:** Minh Nhat Tran, Su-Jin Baek, Hyeong Joon Jun, Sanghun Lee

**Affiliations:** ^1^ Korean Medicine Data Division, Korea Institute of Oriental Medicine, Daejeon, Republic of Korea; ^2^ Korean Convergence Medical Science, University of Science and Technology, Daejeon, Republic of Korea; ^3^ Faculty of Traditional Medicine, Hue University of Medicine and Pharmacy, Hue University, Hue, Vietnam

**Keywords:** bioinformatics, Radix Achyranthis Bidentatae, target organ location, enrichment analysis, gene expression analysis

## Abstract

**Background:** Herbal medicines traditionally target organs for treatment based on medicinal properties, and this theory is widely used for prescriptions. However, the scientific evidence explaining how herbs act on specific organs by biological methods has been still limited. This study used bioinformatic tools to identify the target organ locations of Radix Achyranthis Bidentatae (RAB), a blood-activating herb that nourishes the liver and kidney, strengthens bones, and directs prescription to the lower body.

**Methods:** RAB’s active compounds and targets were collected and predicted using databases such as TCMSP, HIT2.0, and BATMAN-TCM. Next, the RAB’s target list was analyzed based on two approaches to obtain target organ locations. DAVID and Gene ORGANizer enrichment-based approaches were used to enrich an entire gene list, and the BioGPS and HPA gene expression-based approaches were used to analyze the expression of core genes.

**Results:** RAB’s targets were found to be involved in whole blood, blood components, and lymphatic organs across all four tools. Each tool indicated a particular aspect of RAB’s target organ locations: DAVID-enriched genes showed a predominance in blood, liver, and kidneys; Gene ORGANizer showed the effect on low body parts as well as bones and joints; BioGPS and HPA showed high gene expression in bone marrow, lymphoid tissue, and smooth muscle.

**Conclusion:** Our bioinformatics-based target organ location prediction can serve as a modern interpretation tool for the target organ location theory of traditional medicine. Future studies should predict therapeutic target organ locations in complex prescriptions rather than single herbs and conduct experiments to verify predictions.

## 1 Introduction

The rapid growth of biological data, the development of algorithms, and the increase in computer power have made bioinformatics an important contributor to a deeper understanding of existing drugs and to the development of new drugs, in both traditional and modern medicine. Furthermore, herbal medicine-related ‘omics’ data and methods for analyzing molecular mechanisms and biological pathways of herbal medicine have been used to provide innovative ideas (Gu and Chen, 2014). Several bioinformatics-based methods such as network pharmacology, herbal genomics, molecular dynamics simulation, and molecular docking provide knowledge and insight into herbal medicine from different perspectives. The mechanisms underlying herbs and prescriptions have been revealed and further detailed through “multi-target–multi-pathway” paradigms of network pharmacology (Liang et al., 2014; Zhang et al., 2019; Tran and Lee, 2022). These methods are also useful for understanding specific concepts of traditional medicine at the molecular scale such as the synergism of herbal pairs (Wang et al., 2017), or the Qi and Blood-tonifying effects of a group of herbs (Sun et al., 2016; Tran et al., 2022). The common feature of these studies is that the molecular mechanisms were determined using databases to generate large gene sets of interest analyses. Although these types of lists are now frequently produced by biological research, it is still a challenging task to comprehend how gene sets affect an organism’s biology at the tissue and organ level (Brookes and Robinson, 2015).

To overcome this obstacle, multiple tools have been established, allowing researchers to directly or indirectly identify the target organ or tissue locations connected to the genes of interest. For example, Database for Annotation, Visualization and Integrated Discovery (DAVID) is a popular tool that allows lists of genes to be enriched for shared biological pathways, disease associations, as well as tissue expressions (Sherman et al., 2022). Another tool based on gene list enrichment is the Gene ORGANizer, which, unlike DAVID, considers gene–phenotype associations to directly link genes to the human body parts affected by those genes (Gokhman et al., 2017). BRITE (Kanehisa et al., 2016) and Organ System Heterogeneity DB (Mannil et al., 2014) also provide direct linkages between genes and body parts; however, they consider only a few organs and tissues and were not developed for gene list analysis (Gokhman et al., 2017). A further approach for indirectly associating genes to organs is based on expression, which uses mRNA levels to identify the tissues and cell types in which a gene is active, rather than analyzing the enrichment in a list of genes. For instance, the Human Protein Atlas (HPA) is a database that contains localization and expression data for all essential human organs or tissues (Uhlén et al., 2015), and BioGPS provides abundance gene expression data corresponding to tissues or cells based on microarray analyses (Wu et al., 2016). Gene expression is influenced by physiological factors that differ depending on tissue type, and developmental stage. The particular gene expression patterns in organs and tissues provide critical insights regarding gene function (Su et al., 2004; Pan et al., 2013). Therefore, it is critical to assess the tissue mRNA expression patterns of diverse genes at the organ level to investigate the therapeutic effects of herbal target proteins on organs. However, to date, only few studies have used BioGPS to analyze target organ/tissue location for herbs and prescriptions such as *Rhodiola rosea* L. (Zhang X. et al., 2020), Acori Tatarinowii Rhizoma (Song et al., 2018), and Sanhe Decoction (Zhang et al., 2016). Furthermore, the use of BioGPS and the interpretation of its results in these studies remain insufficient. Additionally, other tools that have not been integrated to link herbs to anatomical body parts present an unexplored aspect of this line of research.

Target organ locations, including herbal channel tropism (HCT), is a foundational theory that has influenced traditional treatment for thousands of years (Liu et al., 2013). As per this theory, the therapeutic actions of an herb have selective effects on particular physiological organs or channels (World Health Organization, 2007). Numerous recent studies have demonstrated the value of using systems biology to assess the scientific significance of herbal medicine (Buriani et al., 2012). Radix Achyranthis Bidentatae (RAB), also known as Niuxi, is a blood-activating medicinal herb obtained from the dried roots of *Achyranthes bidentata* Bl. Traditionally, RAB is considered to enter and supplement the liver and kidneys, invigorate blood circulation, and reinforce tendons and bones; directing effects of the prescription to the lower part of the body have also been reported (Chinese Pharmacopoeia Commission, 2015). Research on RAB in modern medicine mostly focused on its pharmacological effects on the bone metabolism, nervous system, and immune system; joint-protection properties; and antioxidation and antitumor effects (He et al., 2017). Using a bioinformatics-based approach, the mechanisms underlying the effects of RAB in the treatment of diseases such as rheumatic arthritis (Fu et al., 2021), osteoarthritis (Zhang et al., 2020), bone trauma (Wu et al., 2021), and breast cancer (Ju et al., 2021) have been elucidated. Nevertheless, the understanding of pathology still differs between traditional and modern medicines, as the terms used for diseases are not identical between the two fields. Specifically, traditional medicine often divides disease models into patterns related to body components or organs, such as Liver and Kidney yin deficiency or Qi and Blood deficiency (Lam et al., 2019). Therefore, principles behind herb-based treatments of diseases are to apply the HCT theory to rebalance body constituents and organ systems. Understanding how multi-organ systems respond to medicinal herbs at a biological system level may assist in the development of improved diagnosis and treatment strategies for complex diseases. However, the application of bioinformatic tools to the association of herbs with organs has not been sufficiently investigated.

**FIGURE 1:**
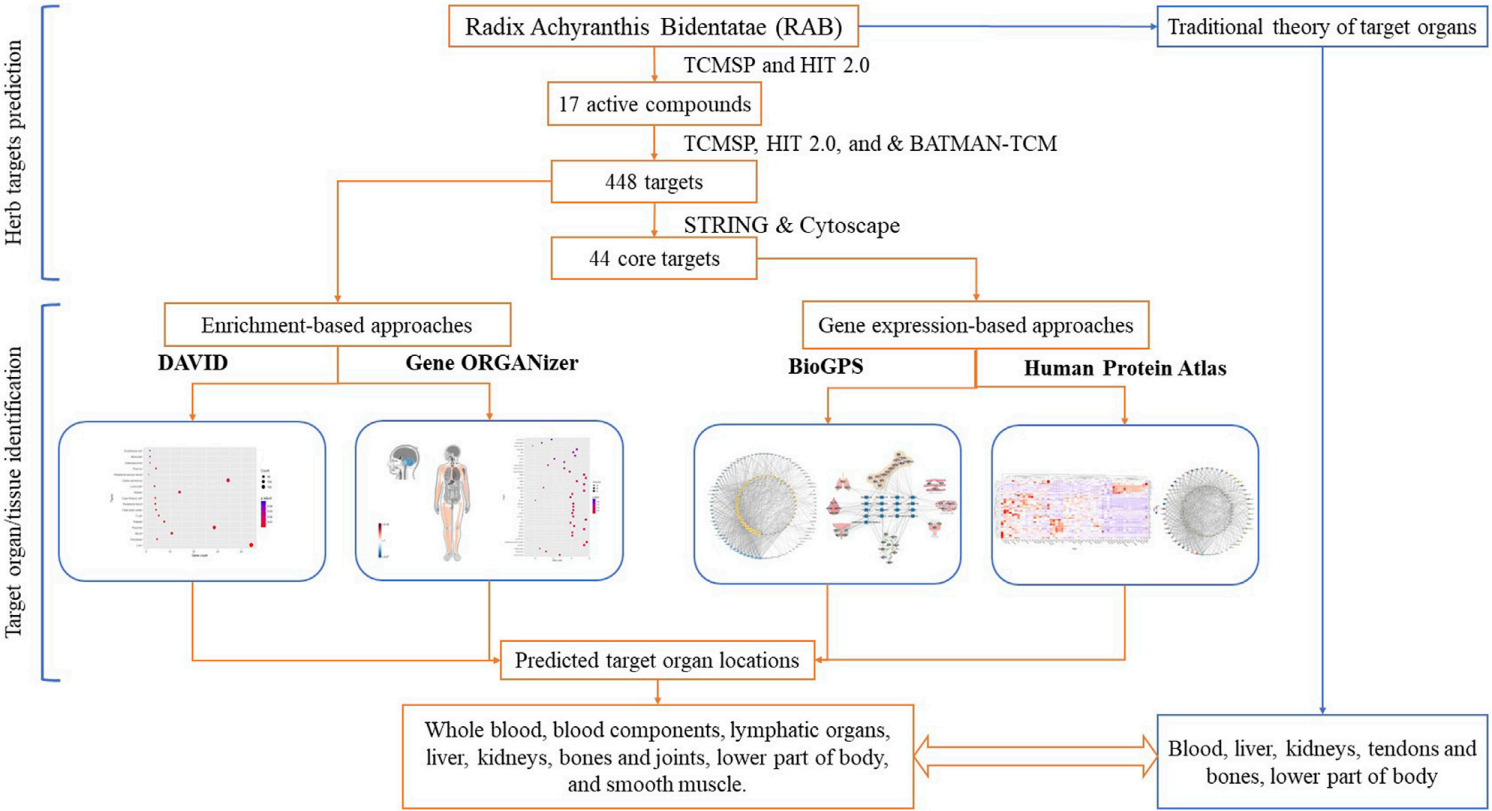
Flow chat of the study.

Therefore, in this study, we used different bioinformatic tools to analyze the target organ/tissue location of herbs, considering RAB as an example. Using gene expression and enrichment-based tools to analyze the targets of RAB, we provided new insight into the HCT mechanisms at the organ/tissue level. The flowchart of this study is shown in Figure 1.

## 2 Methods

### 2.1 Predicting the RAB targets

The RAB targets were predicted in two steps: first, the active compounds were screened, and second, different databases were utilized to predict the targets of the active compounds.

The compounds associated with RAB were retrieved from the Traditional Chinese Medicine Systems Pharmacology Database and Analysis Platform (TCMSP) version 2.3 (Ru et al., 2014) and the Herbal-Ingredient-Target Platform (HIT) version 2.0 (Yan et al., 2022) using “Niuxi” or “Niu Xi” as keywords. Subsequently, the synonym names, Chemical Abstracts Service (CAS), PubChem compound IDs (CID), and International Chemical Identifier (InChIKey) numbers of the compounds were introduced into the PubChem database to obtain the compound structures and ensure that the compounds are recognizable for further steps (Kim et al., 2021). The duplicated compounds between databases or those with no information on the PubChem database were removed from further analysis. Next, to evaluate absorption, distribution, metabolism, and excretion, the oral bioavailability (OB) and drug-likeness (DL) of compounds were obtained from the TCMSP. The active compounds with an OB ≥ 30% and DL ≥ 0.18 were selected (Xu et al., 2012).

Targets of RAB active compounds were obtained from the TCMSP version 2.3 (Ru et al., 2014), HIT version 2.0 (Yan et al., 2022), and Bioinformatics Analysis Tool for Molecular mechANism of TCM (BATMAN-TCM) databases (Liu et al., 2016). From TCMSP, compound-target linkages were acquired via two different approaches: 1) experimentally verified compound-target pairings were obtained from the HIT version 1.0 database; 2) For compounds lacking verified targets, the potential targets were predicted using the SysDT model (Ru et al., 2014). The HIT version 2.0 contains completely updated data compared to HIT version 1.0 by calibrating literature data from 2000 to 2010 and adding experimental data from 2010 to 2020; thus, yielding nearly twice as much data compared to that yielded by the previous version and additional features of target confidence indicators (Yan et al., 2022). The names, CIDs, and CASs of compounds were introduced in the HIT 2.0 database to obtain targets. The targets with levels of A, B, and C were selected for this study. BATMAN-TCM, a similarity-based approach that ranks probable compound-target linkages based on their similarity to the known drug-target interactions, was used to predict potential targets of compounds (Liu et al., 2016). The CIDs for each compound were inputted into the BATMAN-TCM, and the predicted potential targets (including known targets) with a Score_cutoff = 30 were selected. Targets were collected from the three databases. The official gene symbol of the “*Homo sapiens*” genes was obtained from the UniProt database (The UniProt Consortium, 2020), duplicate targets were deleted, and the remaining targets were used for enrichment-based analysis to identify target organ or tissue location.

Core targets were identified by importing all RAB targets into the Search Tool for the Retrieval of Interacting Genes (STRING) database to generate a protein-protein interaction (PPI) network (Szklarczyk et al., 2021). Further, targets were imported into Cytoscape software for topology analysis (Shannon et al., 2003). The PPI network was set up in the STRING database with a high confident interaction score >0.9, *homo sapiens* as species, and FDR stringency = 5%. The disconnected nodes were hidden in the network. Based on the topological analysis in Cytoscape software, targets with degree >2 times the average were selected as core targets. These targets were used for gene expression-based analysis to identify target organ or tissue location.

### 2.2 Linking genes to the organs using enrichment-based analysis approaches

#### 2.2.1 Tissue expression assessment using the DAVID tool

DAVID is a well-known bioinformatics tool that includes a web server as well as a platform for enrichment analyses and functional annotation of gene lists. From the version released in 2006, a gene tissue expression annotation category was added in DAVID and continued to be updated (Huang et al., 2007b). Fisher’s exact test is used in DAVID to calculate the gene enrichment within the annotation categories with EASE score (*p*-value). Importantly, the individual EASE scores were adjusted by multiple testing correction (adjusted *p*-value), such as Bonferroni, Benjamini, and false discovery rate (FDR) tests (Huang et al., 2007a).

In this study, the official names of RAB targets were imported into DAVID (version 2021) to analyze tissue expression annotations, with *Homo sapiens* as species and a *p*-value <0.05 (after Benjamini correction). Uniprot keyword annotations (UP_TISSUE) were chosen for the Tissue expression category. The significant tissues were plotted as a bubble chart using the R package “ggplot2.”

#### 2.2.2 Gene ORGANizer tool

The phenotype-based tool Gene ORGANizer directly connects genes to the bodily parts they influence. It is based on a comprehensive curated database linking more than 7,000 genes to 150 anatomical parts utilizing more than 150,000 gene-organ associations based on DisGeNET and Human Phenotype Ontology databases. These data were converted into relationships between genes and anatomical regions where the phenotype was observed. Overall, the Gene ORGANizer connected 146 different body parts to target genes. The hypergeometric distribution was used in Gene ORGANizer to calculate the significance level of enrichment or depletion, and the mid-range correction was utilized to obtain *p*-values (Gokhman et al., 2017).

In this study, the list of RAB targets was imported into the Gene ORGANizer to analyze gene-organ associations, with a *p*-value <0.05, confident in curation level (inferred from data on humans), typical in frequency of phenotypes (appear in >50% of sick individuals), and FDR in multiple testing corrections. The significant organs and body parts were plotted as a bubble chart using the R package “ggplot2.” The significantly enriched or depleted body parts were visualized as a heat map based on their enrichment or depletion level obtained from the Gene ORGANizer web platform.

### 2.3 Linking genes to the organs using gene expression-based approaches

#### 2.3.1 BioGPS tool

BioGPS is a database for accessing and managing genomic annotation tools. It offers gene expression data from tissues or cells, based on microarray analyses (Wu et al., 2009). BioGPS provides a ‘Gene expression/activity chart’ plugin with a dataset collection function that allows pre-loading of approximately 8,000 datasets from EBI’s ArrayExpress and NCBI’s GEO repositories. These datasets are derived from nine microarray platforms containing data on humans, mice, and rats (Wu et al., 2016).

We constructed the gene-organ localization network using the GeneAtlas U133A gcrma dataset. First, 84 organ tissue samples were used to determine the mRNA expression patterns of each RAB core targets. Second, the overall expression average value across all organs was calculated. Third, genes were linked in the relevant organs where the mRNA expression level was higher than the average. Finally, gene-organ networks were constructed using Cytoscape 3.4.0.

#### 2.3.2 HPA tool

The HPA is a crucial tool for identifying single gene expression patterns in tissue, blood, brain, and cell lines. It contains spatial data regarding the human proteome based on integrated omics methods. This database was generated based on 44 samples from the major tissues and organs of the human body, which were examined using 24,028 antibodies and 16,975 protein-encoding genes, together with RNA-sequencing data for 32 tissues (Uhlén et al., 2015).

In this study, we first downloaded RNA consensus tissue data for RAB core targets. Based on transcriptomics data from HPA (Uhlén et al., 2015) and the Genotype-Tissue Expression (Lonsdale et al., 2013), consensus transcript expression levels per gene were summarized in 54 tissues. The highest transcripts per kilobase million (TPM) value for each gene across the two data sources was used to compute the consensus normalized expression (nTPM) value. The gene expression was then converted to a Z-score for comparing the expression of each gene in different tissues. The R package “ComplexHeatmap” was used to generate a heat map of the core gene expression levels. Lastly, a gene-tissue location network was established by linking the gene to the relevant organs or tissues where it was overexpressed, using Cytoscape. Gene expressions with Z-score >0 were considered to represent overexpression.

## 3 Results

### 3.1 Predicting targets of Radix Achyranthis Bidentatae

By retrieving data from the TCMSP and HIT 2.0 databases, 176 and 13 related compounds were obtained for RAB, respectively. A total of 17 RAB active compounds were screened using OB and DL filtering criteria (Table 1). Beta-sitosterol, β-hydroxyecdysone, and spinasterol were active compounds present in both databases.

**TABLE 1:** List of Radix Achyranthis Bidentatae active compounds.

Molecule name	Pubchem ID	OB (%)	DL	TCMSP ID	HIT2.0 ID
Poriferasta-7,22E-dien-3beta-Ol	5283663	42.98	0.76	MOL001006	
Spinoside A	5281325	41.75	0.4	MOL012537	
Β-ecdysterone	27545171	44.23	0.82	MOL012542	C0653
Berberine	2353	36.86	0.78	MOL001454	
Coptisine	72322	30.67	0.86	MOL001458	
Wogonin	5281703	30.68	0.23	MOL000173	
Delta 7-stigmastenol	12315385	37.42	0.75	MOL002643	
Baicalein	5281605	33.52	0.21	MOL002714	
Baicalin	64982	40.12	0.75	MOL002776	
Epiberberine	160876	43.09	0.78	MOL002897	
Beta-sitosterol	222284	36.91	0.75	MOL000358	C1178
Inophyllum E	455251	38.81	0.85	MOL003847	
Kaempferol	5280863	41.88	0.24	MOL000422	
Spinasterol	5281331	42.98	0.76	MOL004355	C0750
Stigmasterol	5280794	43.83	0.76	MOL000449	
Palmatine	19009	64.6	0.65	MOL000785	
Quercetin	5280343	46.43	0.28	MOL000098	

By entering the information of 17 active compounds into the databases, a total of 448 RAB targets were identified (Supplementary Table S1). Specifically, the TCMSP, HIT2.0, and BATMAN-TCM databases identified 207, 312, and 78 targets, respectively. After removing duplicates, 448 targets were selected. These targets were used for enrichment analyses to identify target organ locations.

**FIGURE 2:**
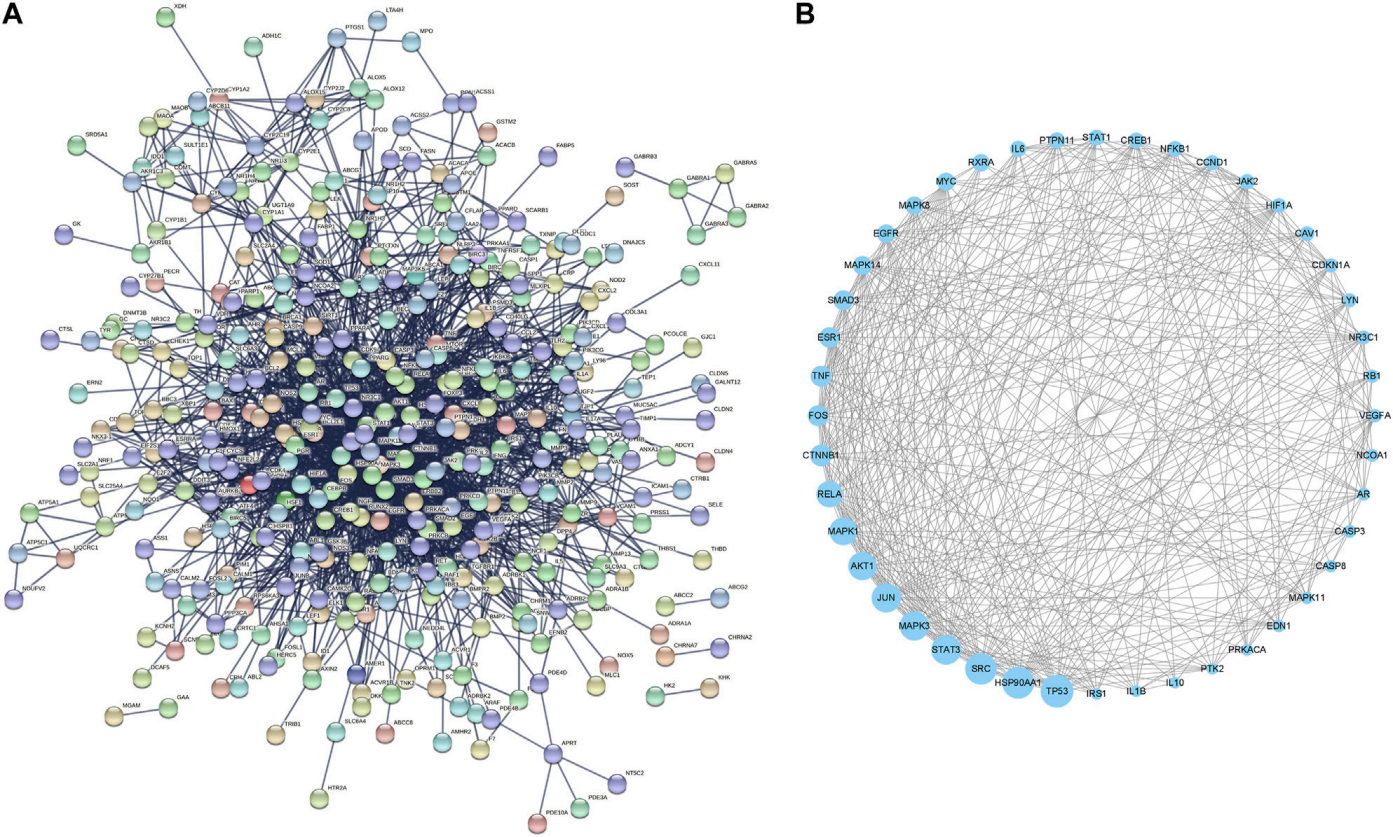
Protein-protein interaction network of **(A)** whole targets and **(B)** core targets of Radix Achyranthis Bidentatae.

Further, the 448 RAB targets were introduced into the STRING database to generate a PPI network (Figure 2A). Topological analysis of this network was performed using Cytoscape, and we identified 44 core targets, which had a degree centrality two-fold higher than the mean value (Figure 2B). Next, these targets were used for gene expression analyses to identify the target organ location.

### 3.2 Linking genes to the organs using enrichment approaches

**FIGURE 3:**
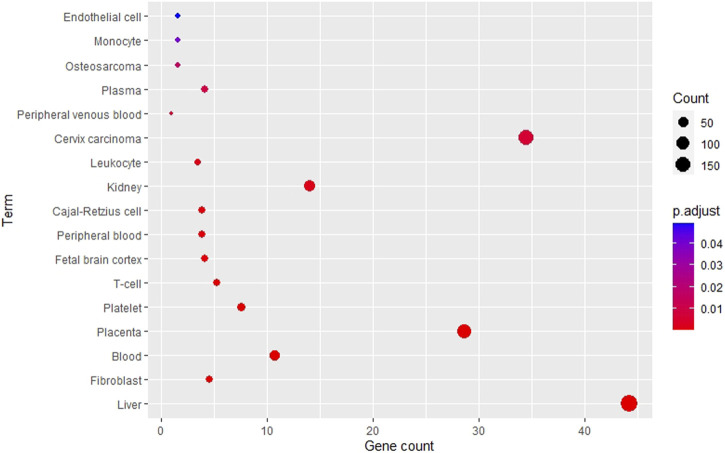
Tissue enrichment analysis of Radix Achyranthis Bidentatae genes using the DAVID tool.

Using the DAVID tool, various tissues were enriched from the RAB target list as shown in Figure 3. The top five most enriched tissues included liver, fibroblast, blood, placenta, and platelets. Interestingly, the liver (*p* = 3.1e-12) was the most enriched organ; along with the kidneys (*p* = 1.4e-3), which are the two channel tropisms of RAB in traditional medicine. In addition, blood (*p* = 1.5e-7) and blood-related components, including platelets (*p* = 5.2e-5), peripheral blood (*p* = 4.6e-4), plasma (*p* = 1.0e-2), and endothelial cells (*p* = 4.9e-2), were also significantly enriched.

**FIGURE 4:**
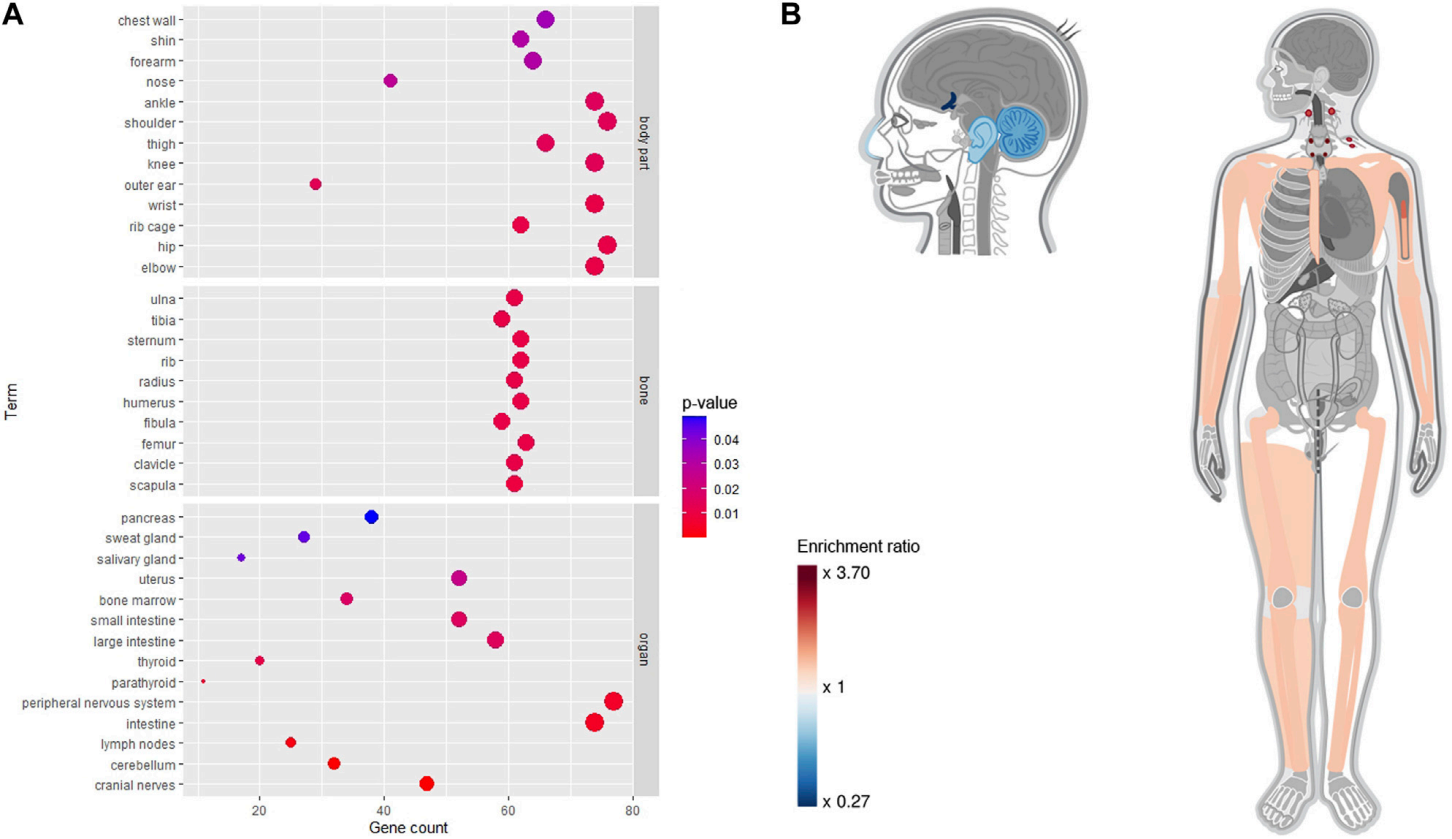
Phenotypical enrichment and depletion analysis for Radix Achyranthis Bidentatae gene using the Gene ORGANizer tool. **(A)** Bubble plot. **(B)** Heat map, with red and blue colors indicating the level of enrichment or depletion, respectively. Gray color indicates non-significant body parts.

Using the Gene ORGANizer, we identified several organs and body parts that exhibited gene enrichment or depletion as shown in Figure 4. In the bubble chart of Figure 4A, the terms were divided into three categories, including organs, bones, and body parts. Cranial nerves, cerebellum, lymph nodes, intestine, and peripheral nervous system were the most significantly affected organs. The results also showed that the RAB targets affect several bones, including scapula, tibia, fibula, and femur. Interestingly, depleted organs or body parts (blue color) were mostly located within the head, specifically cranial nerves (FDR = 2.20e-05), cerebellum (FDR = 5.78e-05), outer ear (FDR = 0.01), and nose (FDR = 0.03); in contrast, the enriched organs and body parts (red color) were mainly distributed in the lower body and limbs, including hip, wrist, elbow (FDR = 0.01), knee, thigh, shoulder (FDR = 0.01), and ankle (FDR = 0.02) (Figures 4A,B). This suggests that RAB targets tend to increase their phenotype expression in lower parts of the body rather than the head.

### 3.3 Linking genes to the organs using gene expression approaches

Specific tissue mRNA expression patterns provide vital insights into gene function. Therefore, it is critical to know the tissue mRNA expression patterns of numerous genes at the organ level to investigate the HCT and traditional effects of RAB. In this study, using gene expression data from the BioGPS and the HPA databases for 44 core targets spread throughout several different tissues and organs, we created target organ location networks to better understand the effects of RAB at the organ- and tissue-level.

**FIGURE 5:**
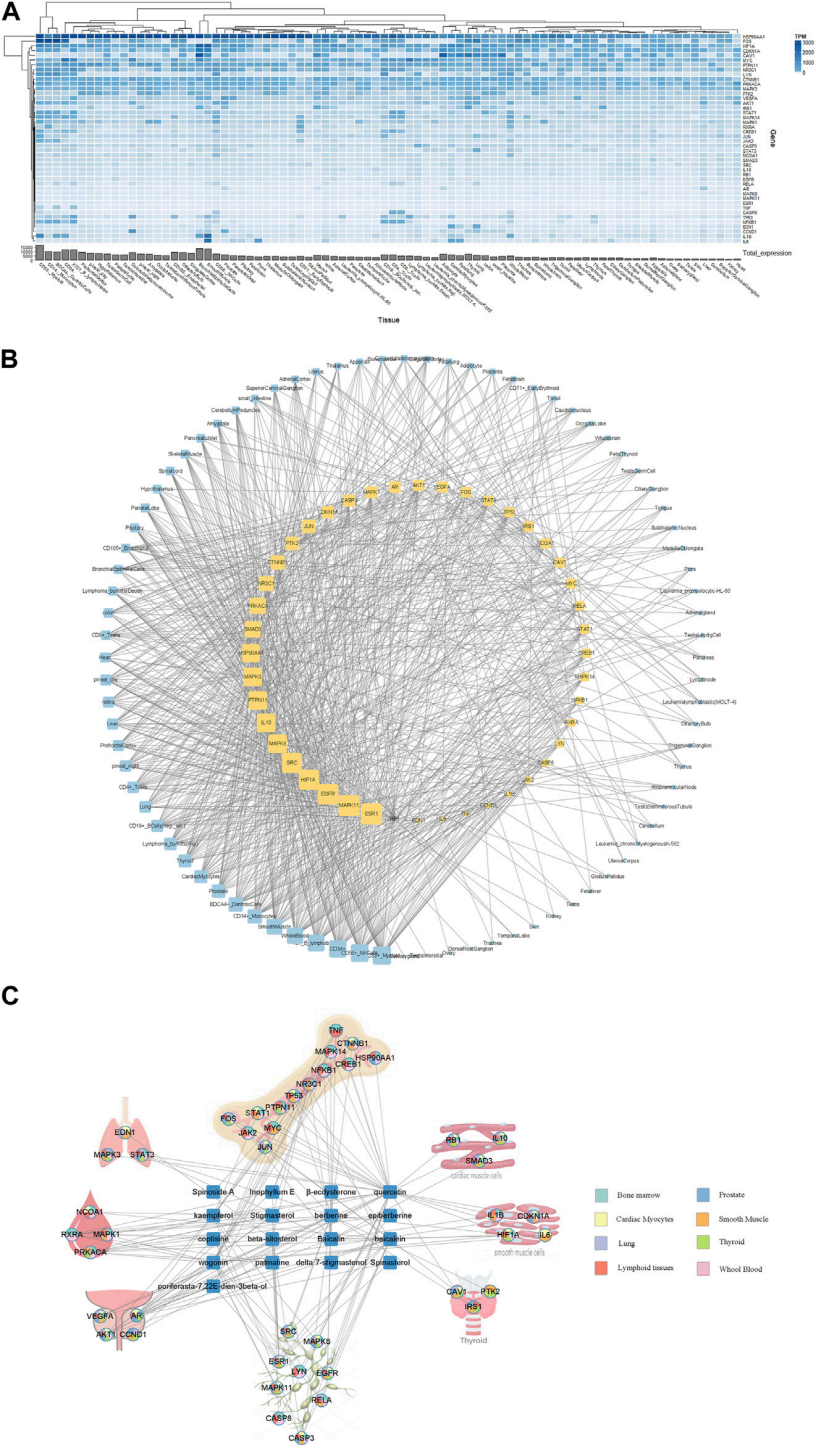
Linking Radix Achyranthis Bidentatae (RAB) targets to organs using the BioGPS tool. **(A)** Core target gene expression. **(B)** Gene-organ network. Yellow and blue nodes represent genes and organs, respectively. Edges represent the gene displayed above average mRNA expressions in each organ, whereas the node size represents the degree centrality value. **(C)** Compound-gene-organ network of most significant organs. The node pie chart illustrates the organs in which each target has a high expression level.

The expression patterns of 44 core targets in 84 normal tissues obtained from the BioGPS database are shown in Figure 5A. These core targets were mostly found in human tissues; however, their mRNA expression levels varied. Networks of RAB targets-organ locations are shown in Figure 5B, where the linkages between genes and organs are represented using the mRNA expressions in each organ. In particular, of 84 organ tissues, CD33^+^ myeloid cells showed the highest number of RAB target overexpression, with 33 of the 44 core targets being overexpressed. This was followed by 32 targets being overexpressed in CD56^+^ natural killer cells, 31 in CD34^+^ hematopoietic stem cells, 30 in B lymphoblasts, 29 in whole blood, 28 in smooth muscle, and 25 in CD14^+^ monocytes and BDCA4^+^ dendritic cells. These results clearly indicate that most targets were overexpressed simultaneously in numerous tissues.

Next, we focused and regrouped on top 15 target organs in the network, including bone marrow (grouped by CD33^+^, CD34^+^, and B lymphoblasts), whole blood (grouped by CD14^+^, CD56^+^, and whole blood), lymphoid tissues (BDCA4^+^, Raji, CD19^+^, and CD4^+^), prostate, smooth muscle, cardiac myocytes, thyroid, and lung. The regrouping was based on cell location (such as lymphoblast, CD14^+^, or CD4^+^) and organ of origin (bone marrow, blood, or lymphoid tissues) during hematopoiesis from stem cells (Orkin and Zon, 2008). RAB targets were linked to the tissues where their mRNA expression levels were the highest. We specifically analyzed the expression profiles of various tissues and discovered that the 14 genes (*TNF, MAPK14, CTNNB1, HSP90AA1, CREB1, NFKB1, NR3C1, TP53, PTPN11, STAT1, MYC, FOS, JAK2*, and *JUN*) showed higher expression levels in the bone marrow than in other tissues, 9 genes (*SRC, MAPK8, ESR1, LYN, EGFR, MAPK11, RELA, CASP8*, and *CASP3*) were lymphoid tissue-specific, 4 were associated with whole blood (*NCOA1, RXRA, MAPK1*, and *PRKACA*), smooth muscle (*IL1B, CDKN1A, HIF1A,* and *IL6*), and the prostate (*VEGFA, AR, AKT1*, and *CCND1*), and 3 were associated with lungs (*EDN1, MAPK3*, and *STAT3*), cardiac myocytes (*RB1, IL10,* and *SMAD3*), and thyroid (*CAV1, PTK2,* and *IRS1*). Based on their expression patterns, 17 active compounds and 44 core targets were organized into an organ location network (Figure 5C).

**FIGURE 6:**
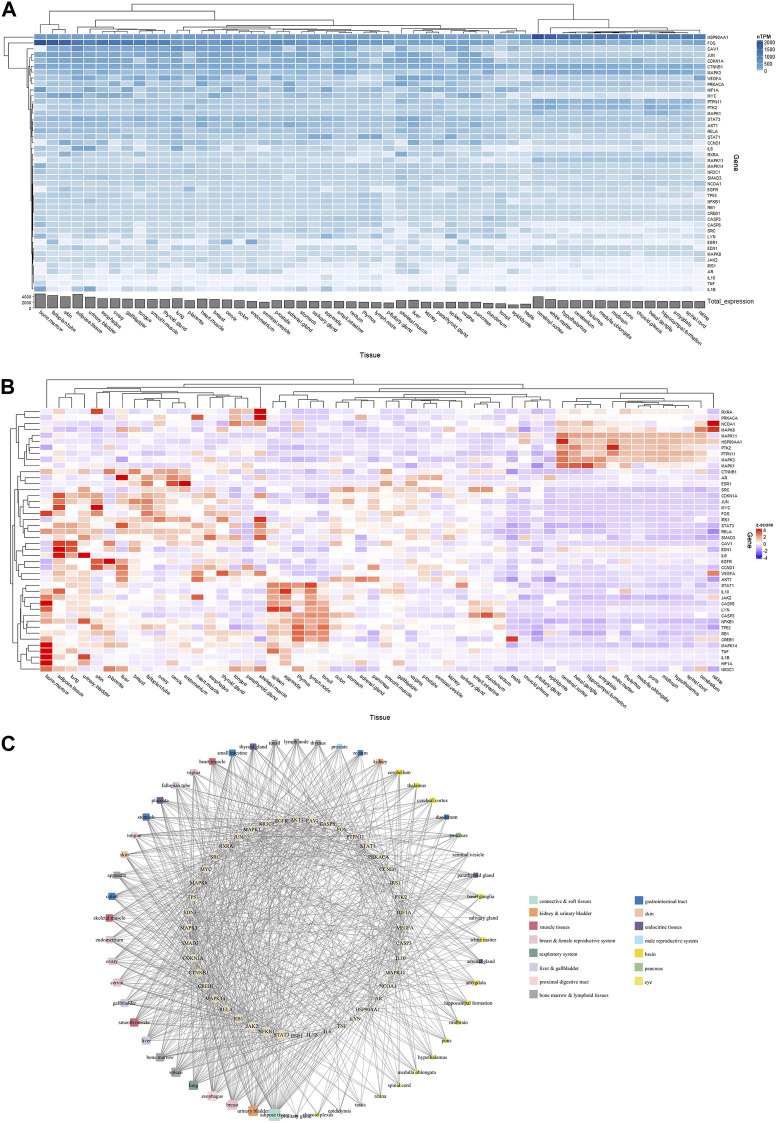
Linking Radix Achyranthis Bidentatae (RAB) targets to the organs using the Human Protein Atlas tool. **(A)** nTPM heatmap and **(B)** Z-score heatmap of core target gene expression. **(C)** Gene-organ network, with ellipse nodes representing target genes, round rectangle nodes representing organ/tissue, and the node size representing the degree centrality value.

As shown in Figure 6A, 44 core targets had their expression profiles mapped onto 54 normal tissues from the HPA database. Core targets had the highest total expression in organs such as bone marrow (4,806.8 nTPM), adipose tissue (4,725.4 nTPM), fallopian tube (4,271.6 nTPM), skin (3,940.5 nTPM), and cerebral cortex (3,936.3 nTPM).

Expression profiles have been converted to Z-scores (Figure 6B), and a network of tissue distribution-potential genes was constructed based on the Z-scores. The network is shown in Figure 6C, including 98 nodes and 860 edges. Each edge represented overexpression (Z-score >0) of the potential gene in a specific tissue or organ. The network analysis results showed that 44 targets were distributed among the adipose tissue (degree = 35), urinary bladder (degree = 28), breast (degree = 28), esophagus (degree = 27), lung and spleen (degree = 26), as well as bone marrow, liver, and smooth muscle (degree = 23). In particular, *STAT3* (degree = 30) and *NFKB1* (degree = 29) were the genes most closely related to organs or tissues.

Ultimately, to obtain a general overview of RAB-related organs following the use of four analytical tools, a comparison of the most affected organs or tissues is described in Table 2.

**TABLE 2:** Top 15 target organ/tissue location of the four tools.

No.	Enrichment-based (sorted by *p*-value)		Expression-based (sorted by network degree)	
	DAVID	Gene ORGANizer	BioGPS	Human Protein Atlas
1	Liver	cranial nerves	CD33^+^ myeloid cells	adipose tissue
2	Fibroblast	cerebellum	CD56^+^ NK cells	breast
3	Blood	lymph nodes	CD34^+^	urinary bladder
4	Placenta	intestine	721 B lymphoblasts	esophagus
5	Platelet	peripheral nervous system	Whole blood	spleen
6	T-cell	parathyroid	Smooth muscle	lung
7	Peripheral blood	thyroid	BDCA4^+^ dendritic cells	bone marrow
8	Fetal brain cortex	large intestine	CD14^+^ monocytes	liver
9	Cajal-Retzius cell	small intestine	Prostate	smooth muscle
10	Kidney	bone marrow	Cardiac Myocytes	cervix
11	Leukocyte	uterus	Thyroid	gallbladder
12	Peripheral venous blood	salivary gland	Lymphoma burkitts (Raji)	ovary
13	Cervix carcinoma	sweat gland	CD19^+^ B Cells (neg._sel.)	endometrium
14	Plasma	pancreas	Lung	skeletal muscle
15	Osteosarcoma	N/A	CD4^+^ T cells	tongue

## 4 Discussion

The target organ location represents the foundation of the traditional theory and describes an herb’s selective effects on specific parts of the body. In traditional medicine, RAB is a blood-activating herb that regulates blood circulation, strengthens bones as well as muscles, and nourishes the liver and kidneys. In prescriptions, it can direct other herbs to have therapeutic effects on the lower part of the body (Chinese Pharmacopoeia Commission, 2015). Classical pharmacological and emerging bioinformatics-based studies have shown that RAB has pharmacological as well as therapeutic effects on various diseases; however, its HCT and effects on anatomical organs have not been adequately investigated. This study revealed the link between RAB and target organ locations by using four different bioinformatic tools. Collectively, RAB targets were associated with whole blood, blood components, and lymphatic organs. Using the DAVID tool, we showed that RAB targets were highly enriched in whole blood, liver, and kidneys. Furthermore, the Gene ORGANizer analysis showed the effects of RAB on lower body parts as well as bones and joints. Alternatively, BioGPS and HPA indicated high RAB-associated gene expression levels in bone marrow, lymphoid tissue, and smooth muscle. Our study revealed the similarities between the target organ locations predicted through bioinformatics and those indicated by traditional medicine.

In this study, we proposed two different approaches to investigate RAB-associated genes: enrichment- and expression-based analyses. Whereas enrichment analyses assess the entire gene list and usually result in target organ locations (e.g., heart), expression analyses show the expression of individual genes and typically concentrate on particular tissues or cell types (e.g., cardiomyocytes) instead of entire organs or systems. Nevertheless, our results still showed a general trend for RAB-associated genes to be associated with whole blood, blood components, and lymphatic organs (Table 2). Previous studies as well as the concept of traditional medicine suggested that RAB has a close relationship with blood, blood circulation, and the immune system. In line with previous findings, the blood-activating feature of RAB might be mediated by the inhibition of blood coagulation and improvements in hemorheological properties (He et al., 2017). A water extract of RAB, for example, was shown to decrease the erythrocyte aggregation index, hematocrit, and whole blood viscosity in wild type rats *in vivo* and significantly prolonged plasma recalcification time, kaolin partial thromboplastin time, and prothrombin time *in vitro* (Li et al., 1990). RAB has been demonstrated to affect the blood rheological property index of an acute blood stasis rat model by significantly lowering the thrombus sizes, the hematocrit, the platelet adhesion rate, the platelet adhesion value, whole blood viscosity, shear relative viscosity, and fibrinogen content (Si et al., 2007). RAB has also been suggested as a potential regulator that affects a variety of immune cells, including natural killer cells, T-lymphocytes, and macrophages (He et al., 2017). In addition, considering whole blood, blood components, and lymphatic organs, each approach explains a distinct aspect of RAB target organ locations.

Enrichment is a common approach for addressing trends within a list of genes, where gene sets are formed based on shared biological or functional properties identified by reference knowledge of the biological domain (Mathur et al., 2018). Our DAVID-based enrichment analysis showed that RAB-associated genes are involved in several blood components such as platelet, peripheral blood, plasma, and endothelial cell (Figure 3). Plasma, with albumin as the predominant protein, is a primary moderator of fluid flow across body compartments and a primary regulator of oncotic pressure in blood vessels (Fanali et al., 2012). The mechanoreceptors of endothelial cells allow them to detect shear stress induced by blood flow across their surface, allowing the blood vessel to adjust its wall thickness and diameter to adapt the blood circulation. Endothelial cells also promote quick responses to brain impulses for blood vessel dilatation by releasing the Nitric oxide, which causes smooth muscle in the vessel wall to relax (Alberts et al., 2002). Alternatively, RAB-associated genes were also highly enriched in the liver and kidney organs, which is in line with the HCT theory and the proclaimed nourishing effect on the liver and kidney. Although the concept of viscera in traditional and modern medicine is not identical, experimental studies clarifying this discrepancy are lacking. Therefore, this gap in knowledge needs to be addressed, and further experiments should be suggested. For the Gene ORGANizer, the list of genes is enriched or depleted against the genome background. Interestingly, for the RAB-associated genes, the head and neck areas were depleted, and the lower body was enriched, with a focus on the bones (Figure 4B); thus, indicating that RAB tends to affect the lower part of the body rather than the upper part. RAB has been previously shown to have an effect on muscles and bones. It can enhance bone strength, and inhibit bone loss by adjusting phosphorus excretion and urinary calcium (He et al., 2010; Zhang et al., 2012), as well as create an environment that is favorable for ossification by increasing blood flow during bone reconstruction (Jiang et al., 2014).

The gene expression-based approach, unlike the enrichment approach that can process the entire gene list, shows the expression of individual genes and places them in a general context. The mRNA expression patterns in tissues provide valuable information for deciphering gene function. In previous studies on target organ locations of herbs, genes with high expression in certain tissues were investigated; however, the selection of cutoff points for high gene expression and subsequent construction of the gene-organ network also varied widely between studies (Wang et al., 2015; Zhang et al., 2016; Zhang et al., 2020; Wang et al., 2020). In this study, we normalized gene expression by mean and Z-score to select genes with high expression. The higher mRNA expression in a tissue compared with the average expression of 84 tissues for each gene was used for analysis using the BioGPS tool (Wang et al., 2015). Furthermore, we selected a cutoff Z-score >0 for the HPA tool. Both HPA and BioGPS showed high expression level for RAB-associated genes in lymphatic organs such as bone marrow and lymphoid tissues. In addition, smooth muscle was also a tissue type with notable RAB-associated gene expression (Figures 5, 6). The primary site of new blood cell production (hemopoiesis) is bone marrow (Lucas, 2021) and it is similar to the role of invigorating herbs in traditional medicine. RAB has a considerable impact on uterine smooth muscle depending on the species and the different physiological conditions (Guo et al., 1997; Yijun et al., 2002). This ties in the role of RAB in the treatment of menstrual pain caused by blood stasis (Ran et al., 2021).

While enrichment and expression-based approaches were shown to be useful for identifying target organ locations in this study, both of them are associated with a number of limitations. First, various tools have different tissue resolutions. For instance, some datasets study the brain as a whole, whereas others study various parts separately (e.g., thalamus, cerebellum, midbrain). Consequently, information on the impact of entire organs is incomplete or skewed. Second, the datasets used have a massive bias towards certain organs (e.g., skin, blood, and brain), whereas other tissues are less explored or unavailable. Third, samples for gene expression analyses are typically collected postmortem, at specific developmental stages, and from selected organ parts. As a result, the collected data rarely accounts for all temporal and structural variations within organs. Fourth, the study was only conducted on a single herb; hence, the results may not reflect the accuracy and objectivity of these approaches. Future studies need to predict target organ locations for multiple herbs in the same herbal group or prescription.

Moreover, the use of bioinformatic tools and databases is always associated with several inherent limitations. For example, prediction of herbal targets by TCMSP and BATMAN-TCM can lead to false positives. Such false positives can introduce incorrect connections in biological networks, like protein-protein interactions; thus, distorting the overall understanding of the biological pathways and leading to inaccurate interpretations. To overcome this limitation, we used two approaches: 1) We combined the TCMSP and BATMAN-TCM databases with HIT 2.0, which includes experimentally verified compound-target pairings (Yan et al., 2022); TCMSP itself also incorporates experimentally validated targets from the HIT 1.0 database (Ru et al., 2014). 2) We used a higher cutoff score (≥30) than the recommended threshold (≥20) for BATMAN-TCM (Liu et al., 2016). Although the PPI network is essential for understanding cell physiology in both normal and diseased states, it has certain drawbacks as the interaction sets obtained from current literature are inadequate (Menche et al., 2015) and biased toward more research protein (Rolland et al., 2014). Future research must use objective methods to examine PPI networks. For example, Guney et al. suggested network-based drug-disease proximity to provide an unbiased assessment of the therapeutic efficacy of a pharmacological protein (Guney et al., 2016).

In conclusion, we used enrichment- and expression-based approaches to detect the target organ locations of RAB using different bioinformatic tools. We discovered that RAB targets were commonly involved in whole blood, blood components, bone marrow, and lymphoid tissue. Furthermore, each tool explained a particular aspect of RAB’s effects on target organ locations: Enriched genes identified using the DAVID tool were observed in whole blood and its components, as well as the liver and kidneys. The Gene ORGANizer showed that the effects of RAB focus on the lower body and impacts bones as well as joints. Lastly, BioGPS and HPA showed high RAB-associated gene expression levels in bone marrow and lymphoid tissue and significant expression levels in smooth muscle. Our study revealed the partial similarities between the RAB target organ locations predicted through bioinformatics and the traditional effects of RAB. Thus, to the best of our knowledge, our study presents the first example for further studies attempting to link genes to target anatomical organs and the HCT theory. In the future, this approach can be extended to predict the target organ location of complex prescriptions rather than individual herbs. Additionally, experimental studies that verify the predicted target organ locations are also necessary.

## Data Availability

The original contributions presented in the study are included in the article/Supplementary Material, further inquiries can be directed to the corresponding author.
