# Supplementary material for: Identifying target organ location of Radix Achyranthis Bidentatae: a bioinformatics approach on active compounds and genes
Source: Front Pharmacol. 2023 Aug 10;14:1187896. doi: 10.3389/fphar.2023.1187896 (PMC10448535; doi:10.3389/fphar.2023.1187896)
Supplement: Supplementary file 1 [file Table1.docx]

Supplementary Material

Identifying target organ location of Radix Achyranthis Bidentatae: A bioinformatics approach on active compounds and genes

# Supplementary Tables

**Table S1.** Gene list of RAB active compound

| **Compound** | **Target gene** | **Database** |
| --- | --- | --- |
| Poriferasta-7,22E-dien-3beta-ol | PGR | TCMSP, BATMAN-TCM |
| Poriferasta-7,22E-dien-3beta-ol | NCOA2 | TCMSP |
| Poriferasta-7,22E-dien-3beta-ol | NR3C2 | TCMSP |
| Poriferasta-7,22E-dien-3beta-ol | TRPV1 | HIT 2.0 |
| Poriferasta-7,22E-dien-3beta-ol | NR1H3 | HIT 2.0 |
| Poriferasta-7,22E-dien-3beta-ol | NR1H2 | HIT 2.0 |
| Poriferasta-7,22E-dien-3beta-ol | ESR1 | BATMAN-TCM |
| Poriferasta-7,22E-dien-3beta-ol | VDR | BATMAN-TCM |
| Poriferasta-7,22E-dien-3beta-ol | CYP27B1 | BATMAN-TCM |
| Poriferasta-7,22E-dien-3beta-ol | GC | BATMAN-TCM |
| Poriferasta-7,22E-dien-3beta-ol | SNW1 | BATMAN-TCM |
| Poriferasta-7,22E-dien-3beta-ol | SRD5A1 | BATMAN-TCM |
| Poriferasta-7,22E-dien-3beta-ol | AR | BATMAN-TCM |
| Poriferasta-7,22E-dien-3beta-ol | NR3C1 | BATMAN-TCM |
| Poriferasta-7,22E-dien-3beta-ol | ANXA1 | BATMAN-TCM |
| Spinoside A | GLRA3 | BATMAN-TCM |
| Spinoside A | NR3C1 | BATMAN-TCM |
| Spinoside A | ATP1A1 | BATMAN-TCM |
| Spinoside A | GABRB3 | BATMAN-TCM |
| β-ecdysterone | EGFR | HIT 2.0 |
| β-ecdysterone | SOD1 | HIT 2.0 |
| β-ecdysterone | MICAL1 | HIT 2.0 |
| β-ecdysterone | CTSL | HIT 2.0 |
| Berberine | NOS2 | TCMSP, HIT 2.0 |
| Berberine | PTGS1 | TCMSP |
| Berberine | KCNH2 | TCMSP |
| Berberine | ESR1 | TCMSP |
| Berberine | AR | TCMSP, HIT 2.0 |
| Berberine | SCN5A | TCMSP |
| Berberine | F10 | TCMSP |
| Berberine | PTGS2 | TCMSP, HIT 2.0 |
| Berberine | NOS3 | TCMSP |
| Berberine | RXRA | TCMSP, HIT 2.0 |
| Berberine | ADRB2 | TCMSP |
| Berberine | HSP90AB1 | TCMSP |
| Berberine | PRKACA | TCMSP |
| Berberine | PRSS1 | TCMSP |
| Berberine | NCOA2 | TCMSP |
| Berberine | PDE10A | TCMSP |
| Berberine | IKBKB | HIT 2.0 |
| Berberine | PRKAA1 | HIT 2.0 |
| Berberine | MTOR | HIT 2.0 |
| Berberine | PPARA | HIT 2.0 |
| Berberine | CYP2E1 | HIT 2.0 |
| Berberine | BACE1 | HIT 2.0 |
| Berberine | SREBF1 | HIT 2.0 |
| Berberine | JUN | HIT 2.0 |
| Berberine | SLC22A2 | HIT 2.0 |
| Berberine | EGFR | HIT 2.0 |
| Berberine | BECN1 | HIT 2.0 |
| Berberine | MAPK14 | HIT 2.0 |
| Berberine | NFE2L2 | HIT 2.0 |
| Berberine | CASP3 | HIT 2.0 |
| Berberine | ACHE | HIT 2.0 |
| Berberine | PTK2 | HIT 2.0 |
| Berberine | CYP2D6 | HIT 2.0 |
| Berberine | SLC22A3 | HIT 2.0 |
| Berberine | STAT3 | HIT 2.0 |
| Berberine | VASP | HIT 2.0 |
| Berberine | TRPV1 | HIT 2.0 |
| Berberine | CYP2J2 | HIT 2.0 |
| Berberine | PPARG | HIT 2.0 |
| Berberine | HSPA5 | HIT 2.0 |
| Berberine | EFNB2 | HIT 2.0 |
| Berberine | ADIPOQ | HIT 2.0 |
| Berberine | MLC1 | HIT 2.0 |
| Berberine | MYLK3 | HIT 2.0 |
| Berberine | SRC | HIT 2.0 |
| Berberine | MMP1 | HIT 2.0 |
| Berberine | MMP9 | HIT 2.0 |
| Berberine | AMER1 | HIT 2.0 |
| Berberine | DNMT1 | HIT 2.0 |
| Berberine | LEP | HIT 2.0 |
| Berberine | PON1 | HIT 2.0 |
| Berberine | IRS1 | HIT 2.0 |
| Berberine | CORT | HIT 2.0 |
| Berberine | CYBB | HIT 2.0 |
| Berberine | AKT1 | HIT 2.0 |
| Berberine | SLC9A3 | HIT 2.0 |
| Berberine | CDH1 | HIT 2.0 |
| Berberine | OLR1 | HIT 2.0 |
| Berberine | SCARB1 | HIT 2.0 |
| Berberine | MIR21 | HIT 2.0 |
| Berberine | ID1 | HIT 2.0 |
| Berberine | CALM3 | HIT 2.0 |
| Berberine | CALM1 | HIT 2.0 |
| Berberine | CALM2 | HIT 2.0 |
| Berberine | HIF1A | HIT 2.0 |
| Berberine | MIR93 | HIT 2.0 |
| Berberine | MIR373 | HIT 2.0 |
| Berberine | EZR | HIT 2.0 |
| Berberine | CTNNB1 | HIT 2.0 |
| Berberine | TIMP1 | HIT 2.0 |
| Berberine | VEGFA | HIT 2.0 |
| Berberine | NFKB1 | HIT 2.0 |
| Berberine | MMP3 | HIT 2.0 |
| Berberine | MMP13 | HIT 2.0 |
| Berberine | PTEN | HIT 2.0 |
| Berberine | DDIT3 | HIT 2.0 |
| Berberine | TP53 | HIT 2.0 |
| Berberine | DNMT3B | HIT 2.0 |
| Berberine | RET | HIT 2.0 |
| Berberine | AQP4 | HIT 2.0 |
| Berberine | ACACB | BATMAN-TCM |
| Berberine | MTAP | BATMAN-TCM |
| Berberine | APRT | BATMAN-TCM |
| Berberine | PECR | BATMAN-TCM |
| Berberine | SRPK2 | BATMAN-TCM |
| Berberine | ACP1 | BATMAN-TCM |
| Coptisine | NOS2 | TCMSP |
| Coptisine | PTGS1 | TCMSP |
| Coptisine | KCNH2 | TCMSP |
| Coptisine | ESR1 | TCMSP |
| Coptisine | AR | TCMSP |
| Coptisine | SCN5A | TCMSP |
| Coptisine | PTGS2 | TCMSP |
| Coptisine | NOS3 | TCMSP |
| Coptisine | PRSS1 | TCMSP |
| Coptisine | CASP1 | HIT 2.0 |
| Coptisine | IDO1 | HIT 2.0 |
| Coptisine | DHODH | HIT 2.0 |
| Coptisine | MAOA | HIT 2.0 |
| Coptisine | MMP9 | HIT 2.0 |
| Coptisine | TIMP1 | HIT 2.0 |
| Coptisine | MAPK1 | HIT 2.0 |
| Wogonin | NOS2 | TCMSP, HIT 2.0 |
| Wogonin | PTGS1 | TCMSP, HIT 2.0 |
| Wogonin | ESR1 | TCMSP |
| Wogonin | AR | TCMSP |
| Wogonin | SCN5A | TCMSP |
| Wogonin | PPARG | TCMSP |
| Wogonin | PTGS2 | TCMSP, HIT 2.0 |
| Wogonin | RXRA | TCMSP |
| Wogonin | PDE3A | TCMSP |
| Wogonin | DPP4 | TCMSP |
| Wogonin | MAPK14 | TCMSP |
| Wogonin | GSK3B | TCMSP, HIT 2.0 |
| Wogonin | HSP90AB1 | TCMSP |
| Wogonin | CDK2 | TCMSP |
| Wogonin | PIK3CG | TCMSP |
| Wogonin | CHEK1 | TCMSP |
| Wogonin | PRKACA | TCMSP |
| Wogonin | PRSS1 | TCMSP |
| Wogonin | ADRB2 | TCMSP |
| Wogonin | GABRA1 | TCMSP |
| Wogonin | RELA | TCMSP, HIT 2.0 |
| Wogonin | AKT1 | TCMSP, HIT 2.0 |
| Wogonin | CCND1 | TCMSP, HIT 2.0 |
| Wogonin | BCL2 | TCMSP, HIT 2.0 |
| Wogonin | CDKN3 | TCMSP |
| Wogonin | EIF6 | TCMSP |
| Wogonin | BAX | TCMSP, HIT 2.0 |
| Wogonin | CASP9 | TCMSP |
| Wogonin | KDR | TCMSP, HIT 2.0 |
| Wogonin | TNF | TCMSP |
| Wogonin | JUNB | TCMSP |
| Wogonin | IL6 | TCMSP |
| Wogonin | AHSA1 | TCMSP |
| Wogonin | CASP3 | TCMSP, HIT 2.0 |
| Wogonin | TP53 | TCMSP, HIT 2.0 |
| Wogonin | BBC3 | TCMSP, HIT 2.0 |
| Wogonin | TEP1 | TCMSP |
| Wogonin | MMP1 | TCMSP |
| Wogonin | XCL2 | TCMSP |
| Wogonin | PRKCD | TCMSP, HIT 2.0 |
| Wogonin | PTGER3 | TCMSP |
| Wogonin | FN1 | TCMSP, HIT 2.0 |
| Wogonin | CXCL8 | TCMSP, HIT 2.0 |
| Wogonin | MCL1 | TCMSP, HIT 2.0 |
| Wogonin | CYP1A2 | HIT 2.0 |
| Wogonin | HIF1A | HIT 2.0 |
| Wogonin | CTNNB1 | HIT 2.0 |
| Wogonin | MMP13 | HIT 2.0 |
| Wogonin | NFKB1 | HIT 2.0 |
| Wogonin | MUC5AC | HIT 2.0 |
| Wogonin | CYP2C19 | HIT 2.0 |
| Wogonin | MMP1 | HIT 2.0 |
| Wogonin | CCL2 | HIT 2.0 |
| Wogonin | CDK9 | HIT 2.0 |
| Wogonin | CDKN1A | HIT 2.0 |
| Wogonin | IL6 | HIT 2.0 |
| Wogonin | JUN | HIT 2.0 |
| Wogonin | F3 | HIT 2.0 |
| Wogonin | CCL26 | HIT 2.0 |
| Wogonin | MMP9 | HIT 2.0 |
| Wogonin | CDKN1B | HIT 2.0 |
| Wogonin | MAPK11 | HIT 2.0 |
| Wogonin | TNF | HIT 2.0 |
| Wogonin | ERN1 | HIT 2.0 |
| Wogonin | VEGFA | HIT 2.0 |
| Wogonin | CFLAR | HIT 2.0 |
| Wogonin | EGFR | HIT 2.0 |
| Wogonin | SPP1 | HIT 2.0 |
| Wogonin | NEDD4L | HIT 2.0 |
| Wogonin | CDK4 | HIT 2.0 |
| Wogonin | TRAF2 | HIT 2.0 |
| Wogonin | MAPK1 | HIT 2.0 |
| Delta 7-stigmastenol | PGR | TCMSP |
| Baicalein | PTGS1 | TCMSP |
| Baicalein | AR | TCMSP, HIT 2.0 |
| Baicalein | PTGS2 | TCMSP, HIT 2.0 |
| Baicalein | HSP90AB1 | TCMSP |
| Baicalein | PRKACA | TCMSP |
| Baicalein | DPP4 | TCMSP |
| Baicalein | PIK3CG | TCMSP, HIT 2.0 |
| Baicalein | PDE3A | TCMSP |
| Baicalein | PRSS1 | TCMSP |
| Baicalein | NCOA2 | TCMSP |
| Baicalein | NCOA1 | TCMSP |
| Baicalein | RELA | TCMSP, HIT 2.0 |
| Baicalein | AKT1 | TCMSP, HIT 2.0 |
| Baicalein | VEGFA | TCMSP, HIT 2.0 |
| Baicalein | BCL2 | TCMSP, HIT 2.0 |
| Baicalein | FOS | TCMSP, HIT 2.0 |
| Baicalein | BAX | TCMSP, HIT 2.0 |
| Baicalein | MMP9 | TCMSP, HIT 2.0 |
| Baicalein | CASP3 | TCMSP, HIT 2.0 |
| Baicalein | TP53 | TCMSP, HIT 2.0 |
| Baicalein | HIF1A | TCMSP, HIT 2.0 |
| Baicalein | FOSL1 | TCMSP, HIT 2.0 |
| Baicalein | FOSL2 | TCMSP, HIT 2.0 |
| Baicalein | CDK1 | TCMSP, HIT 2.0 |
| Baicalein | CCNB1 | TCMSP, HIT 2.0 |
| Baicalein | MPO | TCMSP, HIT 2.0 |
| Baicalein | AHR | TCMSP, HIT 2.0 |
| Baicalein | IGF2 | TCMSP, HIT 2.0 |
| Baicalein | ALOX12 | TCMSP, HIT 2.0 |
| Baicalein | NFATC1 | TCMSP, HIT 2.0 |
| Baicalein | TDRD7 | TCMSP |
| Baicalein | EGLN1 | TCMSP, HIT 2.0 |
| Baicalein | NOX5 | TCMSP, HIT 2.0 |
| Baicalein | FABP5 | TCMSP, HIT 2.0 |
| Baicalein | APOD | TCMSP, HIT 2.0 |
| Baicalein | NOS2 | HIT 2.0 |
| Baicalein | ALOX15 | HIT 2.0 |
| Baicalein | PIK3CD | HIT 2.0 |
| Baicalein | PIK3CB | HIT 2.0 |
| Baicalein | GAA | HIT 2.0 |
| Baicalein | CXADR | HIT 2.0 |
| Baicalein | TYR | HIT 2.0 |
| Baicalein | NR1I3 | HIT 2.0 |
| Baicalein | MAPK1 | HIT 2.0 |
| Baicalein | TGFBR1 | HIT 2.0 |
| Baicalein | GUSB | HIT 2.0 |
| Baicalein | LY96 | HIT 2.0 |
| Baicalein | ABCB1 | HIT 2.0 |
| Baicalein | CYCS | HIT 2.0 |
| Baicalein | ESR1 | HIT 2.0 |
| Baicalein | JUN | HIT 2.0 |
| Baicalein | SRC | HIT 2.0 |
| Baicalein | HMOX1 | HIT 2.0 |
| Baicalein | EGFR | HIT 2.0 |
| Baicalein | CYP3A4 | HIT 2.0 |
| Baicalein | CAV1 | HIT 2.0 |
| Baicalein | CRTC1 | HIT 2.0 |
| Baicalein | COMT | HIT 2.0 |
| Baicalein | NFE2L2 | HIT 2.0 |
| Baicalein | MTOR | HIT 2.0 |
| Baicalein | MAOB | HIT 2.0 |
| Baicalein | MMP2 | HIT 2.0 |
| Baicalin | F10 | TCMSP |
| Baicalin | PTPN1 | TCMSP |
| Baicalin | IL6 | HIT 2.0 |
| Baicalin | ALOX5 | HIT 2.0 |
| Baicalin | MMP9 | HIT 2.0 |
| Baicalin | FN1 | HIT 2.0 |
| Baicalin | JAK2 | HIT 2.0 |
| Baicalin | TYR | HIT 2.0 |
| Baicalin | SOST | HIT 2.0 |
| Baicalin | PPARG | HIT 2.0 |
| Baicalin | CYP3A | HIT 2.0 |
| Baicalin | TLR2 | HIT 2.0 |
| Baicalin | TLR4 | HIT 2.0 |
| Baicalin | NOD2 | HIT 2.0 |
| Baicalin | SMAD2 | HIT 2.0 |
| Baicalin | SMAD3 | HIT 2.0 |
| Baicalin | KDM1A | HIT 2.0 |
| Baicalin | NEU1 | HIT 2.0 |
| Baicalin | CAMK2G | HIT 2.0 |
| Baicalin | IL1B | HIT 2.0 |
| Baicalin | TNF | HIT 2.0 |
| Baicalin | CCN2 | HIT 2.0 |
| Baicalin | STAT3 | HIT 2.0 |
| Baicalin | SOD1 | HIT 2.0 |
| Baicalin | CYP1A2 | HIT 2.0 |
| Baicalin | IL17A | HIT 2.0 |
| Baicalin | BAX | HIT 2.0 |
| Baicalin | BCL2 | HIT 2.0 |
| Baicalin | FABP1 | HIT 2.0 |
| Baicalin | MEG3 | HIT 2.0 |
| Baicalin | TNFRSF11B | HIT 2.0 |
| Baicalin | NR3C1 | HIT 2.0 |
| Baicalin | SCNN1A | HIT 2.0 |
| Baicalin | ADORA2A | HIT 2.0 |
| Baicalin | NR1H3 | HIT 2.0 |
| Baicalin | FOXP3 | HIT 2.0 |
| Baicalin | GUSB | HIT 2.0 |
| Baicalin | DKK1 | HIT 2.0 |
| Baicalin | GSK3B | HIT 2.0 |
| Epiberberine | NOS2 | TCMSP |
| Epiberberine | KCNH2 | TCMSP |
| Epiberberine | ESR1 | TCMSP |
| Epiberberine | AR | TCMSP |
| Epiberberine | PTGS2 | TCMSP |
| Epiberberine | NOS3 | TCMSP |
| Epiberberine | RXRA | TCMSP |
| Epiberberine | PRKACA | TCMSP |
| Epiberberine | PRSS1 | TCMSP |
| Epiberberine | NCOA2 | TCMSP |
| Epiberberine | PDE10A | TCMSP |
| Beta-sitosterol | PGR | TCMSP, BATMAN-TCM |
| Beta-sitosterol | NCOA2 | TCMSP |
| Beta-sitosterol | PTGS1 | TCMSP |
| Beta-sitosterol | PTGS2 | TCMSP |
| Beta-sitosterol | HSP90AB1 | TCMSP |
| Beta-sitosterol | PIK3CG | TCMSP |
| Beta-sitosterol | KCNH2 | TCMSP |
| Beta-sitosterol | PRKACA | TCMSP |
| Beta-sitosterol | DRD1 | TCMSP |
| Beta-sitosterol | CHRM3 | TCMSP |
| Beta-sitosterol | CHRM1 | TCMSP |
| Beta-sitosterol | SCN5A | TCMSP |
| Beta-sitosterol | GABRA2 | TCMSP |
| Beta-sitosterol | CHRM4 | TCMSP |
| Beta-sitosterol | PDE3A | TCMSP |
| Beta-sitosterol | HTR2A | TCMSP |
| Beta-sitosterol | GABRA5 | TCMSP |
| Beta-sitosterol | ADRA1A | TCMSP |
| Beta-sitosterol | GABRA3 | TCMSP |
| Beta-sitosterol | CHRM2 | TCMSP |
| Beta-sitosterol | ADRA1B | TCMSP |
| Beta-sitosterol | ADRB2 | TCMSP |
| Beta-sitosterol | CHRNA2 | TCMSP |
| Beta-sitosterol | SLC6A4 | TCMSP |
| Beta-sitosterol | OPRM1 | TCMSP |
| Beta-sitosterol | GABRA1 | TCMSP |
| Beta-sitosterol | CHRNA7 | TCMSP |
| Beta-sitosterol | BCL2 | TCMSP, HIT 2.0 |
| Beta-sitosterol | BAX | TCMSP, HIT 2.0 |
| Beta-sitosterol | CASP9 | TCMSP, HIT 2.0 |
| Beta-sitosterol | JUNB | TCMSP |
| Beta-sitosterol | CASP3 | TCMSP, HIT 2.0 |
| Beta-sitosterol | CASP8 | TCMSP, HIT 2.0 |
| Beta-sitosterol | PRKCA | TCMSP, HIT 2.0 |
| Beta-sitosterol | TGFB1 | TCMSP, HIT 2.0 |
| Beta-sitosterol | PON1 | TCMSP |
| Beta-sitosterol | MAP2 | TCMSP, HIT 2.0 |
| Beta-sitosterol | PRKAA2 | HIT 2.0 |
| Beta-sitosterol | EDN1 | HIT 2.0 |
| Beta-sitosterol | SOAT1 | BATMAN-TCM |
| Beta-sitosterol | ANPEP | BATMAN-TCM |
| Beta-sitosterol | NPC1L1 | BATMAN-TCM |
| Beta-sitosterol | ESR1 | BATMAN-TCM |
| Beta-sitosterol | NR1H4 | BATMAN-TCM |
| Beta-sitosterol | VDR | BATMAN-TCM |
| Beta-sitosterol | CYP27B1 | BATMAN-TCM |
| Beta-sitosterol | GC | BATMAN-TCM |
| Beta-sitosterol | SNW1 | BATMAN-TCM |
| Beta-sitosterol | AR | BATMAN-TCM |
| Beta-sitosterol | NR3C1 | BATMAN-TCM |
| Inophyllum E | PTGS1 | TCMSP |
| Inophyllum E | ESR1 | TCMSP |
| Inophyllum E | AR | TCMSP |
| Inophyllum E | F10 | TCMSP |
| Inophyllum E | PTGS2 | TCMSP |
| Inophyllum E | TOP2A | TCMSP |
| Inophyllum E | ESR2 | TCMSP |
| Inophyllum E | GSK3B | TCMSP |
| Inophyllum E | PIM1 | TCMSP |
| Inophyllum E | NFKB1 | BATMAN-TCM |
| Inophyllum E | CYSLTR1 | BATMAN-TCM |
| Inophyllum E | TNF | BATMAN-TCM |
| Inophyllum E | RNASE3 | BATMAN-TCM |
| Inophyllum E | MUC2 | BATMAN-TCM |
| Inophyllum E | IL5 | BATMAN-TCM |
| Inophyllum E | TRIB1 | BATMAN-TCM |
| Inophyllum E | SDCBP | BATMAN-TCM |
| Inophyllum E | LTA | BATMAN-TCM |
| Inophyllum E | RXRA | BATMAN-TCM |
| Kaempferol | NOS2 | TCMSP, HIT 2.0 |
| Kaempferol | PTGS1 | TCMSP |
| Kaempferol | AR | TCMSP |
| Kaempferol | PPARG | TCMSP, HIT 2.0 |
| Kaempferol | PTGS2 | TCMSP, HIT 2.0 |
| Kaempferol | HSP90AB1 | TCMSP |
| Kaempferol | PIK3CG | TCMSP |
| Kaempferol | PRKACA | TCMSP |
| Kaempferol | NCOA2 | TCMSP |
| Kaempferol | DPP4 | TCMSP |
| Kaempferol | PRSS1 | TCMSP |
| Kaempferol | PGR | TCMSP |
| Kaempferol | F2 | TCMSP |
| Kaempferol | CHRM1 | TCMSP |
| Kaempferol | NOS3 | TCMSP |
| Kaempferol | GABRA2 | TCMSP |
| Kaempferol | ACHE | TCMSP |
| Kaempferol | SLC6A2 | TCMSP |
| Kaempferol | CHRM2 | TCMSP |
| Kaempferol | ADRA1B | TCMSP |
| Kaempferol | GABRA1 | TCMSP |
| Kaempferol | TOP2A | TCMSP |
| Kaempferol | F7 | TCMSP |
| Kaempferol | RELA | TCMSP |
| Kaempferol | IKBKB | TCMSP |
| Kaempferol | AKT1 | TCMSP |
| Kaempferol | BCL2 | TCMSP, HIT 2.0 |
| Kaempferol | BAX | TCMSP, HIT 2.0 |
| Kaempferol | TNF | TCMSP |
| Kaempferol | JUNB | TCMSP |
| Kaempferol | AHSA1 | TCMSP |
| Kaempferol | CASP3 | TCMSP, HIT 2.0 |
| Kaempferol | MAPK8 | TCMSP, HIT 2.0 |
| Kaempferol | XDH | TCMSP, HIT 2.0 |
| Kaempferol | MMP1 | TCMSP |
| Kaempferol | STAT1 | TCMSP, HIT 2.0 |
| Kaempferol | CDK1 | TCMSP, HIT 2.0 |
| Kaempferol | HMOX1 | TCMSP, HIT 2.0 |
| Kaempferol | CYP3A4 | TCMSP |
| Kaempferol | CYP1A2 | TCMSP, HIT 2.0 |
| Kaempferol | CYP1A1 | TCMSP, HIT 2.0 |
| Kaempferol | ICAM1 | TCMSP, HIT 2.0 |
| Kaempferol | SELE | TCMSP, HIT 2.0 |
| Kaempferol | VCAM1 | TCMSP, HIT 2.0 |
| Kaempferol | NR1I2 | TCMSP, HIT 2.0 |
| Kaempferol | CYP1B1 | TCMSP, HIT 2.0 |
| Kaempferol | ALOX5 | TCMSP |
| Kaempferol | HAS2 | TCMSP |
| Kaempferol | GSTP1 | TCMSP |
| Kaempferol | AHR | TCMSP, HIT 2.0 |
| Kaempferol | PSMD3 | TCMSP |
| Kaempferol | SLC2A4 | TCMSP |
| Kaempferol | NR1I3 | TCMSP, HIT 2.0 |
| Kaempferol | INSR | TCMSP |
| Kaempferol | DIO1 | TCMSP, HIT 2.0 |
| Kaempferol | PPP3CA | TCMSP |
| Kaempferol | GSTM1 | TCMSP, HIT 2.0 |
| Kaempferol | GSTM2 | TCMSP, HIT 2.0 |
| Kaempferol | AKR1C3 | TCMSP |
| Kaempferol | SLPI | TCMSP |
| Kaempferol | MMP1 | HIT 2.0 |
| Kaempferol | TNF | HIT 2.0 |
| Kaempferol | BIRC2 | HIT 2.0 |
| Kaempferol | BIRC3 | HIT 2.0 |
| Kaempferol | NFKBIA | HIT 2.0 |
| Kaempferol | MMP9 | HIT 2.0 |
| Kaempferol | MCU | HIT 2.0 |
| Kaempferol | NFKB1 | HIT 2.0 |
| Kaempferol | JUN | HIT 2.0 |
| Kaempferol | PLEK | HIT 2.0 |
| Kaempferol | NSFL1C | HIT 2.0 |
| Kaempferol | ESRRA | HIT 2.0 |
| Kaempferol | FOXP3 | HIT 2.0 |
| Kaempferol | TJP1 | HIT 2.0 |
| Kaempferol | MAPK14 | HIT 2.0 |
| Kaempferol | CXCL8 | HIT 2.0 |
| Kaempferol | BMPR2 | HIT 2.0 |
| Kaempferol | MIR21 | HIT 2.0 |
| Kaempferol | RPS6KA3 | HIT 2.0 |
| Kaempferol | CLDN2 | HIT 2.0 |
| Kaempferol | UGT3A1 | BATMAN-TCM |
| Spinasterol | PGR | TCMSP, BATMAN-TCM |
| Spinasterol | NR3C2 | TCMSP |
| Spinasterol | NCOA2 | TCMSP |
| Spinasterol | TRPV1 | HIT 2.0 |
| Spinasterol | NR1H3 | HIT 2.0 |
| Spinasterol | NR1H2 | HIT 2.0 |
| Spinasterol | ESR1 | BATMAN-TCM |
| Spinasterol | VDR | BATMAN-TCM |
| Spinasterol | CYP27B1 | BATMAN-TCM |
| Spinasterol | GC | BATMAN-TCM |
| Spinasterol | SNW1 | BATMAN-TCM |
| Spinasterol | SRD5A1 | BATMAN-TCM |
| Spinasterol | AR | BATMAN-TCM |
| Spinasterol | NR3C1 | BATMAN-TCM |
| Spinasterol | ANXA1 | BATMAN-TCM |
| Stigmasterol | PGR | TCMSP, BATMAN-TCM |
| Stigmasterol | NR3C2 | TCMSP |
| Stigmasterol | NCOA2 | TCMSP |
| Stigmasterol | ADH1C | TCMSP |
| Stigmasterol | IGHG1 | TCMSP |
| Stigmasterol | RXRA | TCMSP |
| Stigmasterol | NCOA1 | TCMSP |
| Stigmasterol | PTGS1 | TCMSP |
| Stigmasterol | PTGS2 | TCMSP |
| Stigmasterol | ADRA2A | TCMSP |
| Stigmasterol | SLC6A2 | TCMSP |
| Stigmasterol | SLC6A3 | TCMSP |
| Stigmasterol | ADRB2 | TCMSP |
| Stigmasterol | AKR1B1 | TCMSP |
| Stigmasterol | PLAU | TCMSP |
| Stigmasterol | LTA4H | TCMSP |
| Stigmasterol | MAOB | TCMSP |
| Stigmasterol | MAOA | TCMSP |
| Stigmasterol | PRKACA | TCMSP |
| Stigmasterol | CTRB1 | TCMSP |
| Stigmasterol | CHRM3 | TCMSP |
| Stigmasterol | CHRM1 | TCMSP |
| Stigmasterol | ADRB1 | TCMSP |
| Stigmasterol | SCN5A | TCMSP |
| Stigmasterol | HTR2A | TCMSP |
| Stigmasterol | ADRA1A | TCMSP |
| Stigmasterol | GABRA3 | TCMSP |
| Stigmasterol | CHRM2 | TCMSP |
| Stigmasterol | ADRA1B | TCMSP |
| Stigmasterol | GABRA1 | TCMSP |
| Stigmasterol | CHRNA7 | TCMSP |
| Stigmasterol | NR1H2 | HIT 2.0 |
| Stigmasterol | SOAT1 | BATMAN-TCM |
| Stigmasterol | ANPEP | BATMAN-TCM |
| Stigmasterol | NPC1L1 | BATMAN-TCM |
| Stigmasterol | ESR1 | BATMAN-TCM |
| Stigmasterol | ABCC2 | BATMAN-TCM |
| Stigmasterol | AKT1 | BATMAN-TCM |
| Stigmasterol | ACVR1 | BATMAN-TCM |
| Stigmasterol | ASNA1 | BATMAN-TCM |
| Stigmasterol | ADRBK2 | BATMAN-TCM |
| Stigmasterol | ACSS2 | BATMAN-TCM |
| Stigmasterol | ACVRL1 | BATMAN-TCM |
| Stigmasterol | ADCY1 | BATMAN-TCM |
| Stigmasterol | NT5C2 | BATMAN-TCM |
| Stigmasterol | AMHR2 | BATMAN-TCM |
| Stigmasterol | TNK2 | BATMAN-TCM |
| Stigmasterol | ASNS | BATMAN-TCM |
| Stigmasterol | ABL2 | BATMAN-TCM |
| Stigmasterol | ADRBK1 | BATMAN-TCM |
| Stigmasterol | PRKAA1 | BATMAN-TCM |
| Stigmasterol | ABCC9 | BATMAN-TCM |
| Stigmasterol | ACSS1 | BATMAN-TCM |
| Stigmasterol | ABL1 | BATMAN-TCM |
| Stigmasterol | AFG3L2 | BATMAN-TCM |
| Stigmasterol | CDK15 | BATMAN-TCM |
| Stigmasterol | ASS1 | BATMAN-TCM |
| Stigmasterol | ACVR1B | BATMAN-TCM |
| Stigmasterol | ACSL1 | BATMAN-TCM |
| Stigmasterol | ABCA1 | BATMAN-TCM |
| Stigmasterol | NAE1 | BATMAN-TCM |
| Stigmasterol | APAF1 | BATMAN-TCM |
| Stigmasterol | ABCC8 | BATMAN-TCM |
| Stigmasterol | ALK | BATMAN-TCM |
| Stigmasterol | ABCB11 | BATMAN-TCM |
| Stigmasterol | ABCB1 | BATMAN-TCM |
| Stigmasterol | ABCG1 | BATMAN-TCM |
| Stigmasterol | ARAF | BATMAN-TCM |
| Stigmasterol | SLC25A4 | BATMAN-TCM |
| Stigmasterol | VDR | BATMAN-TCM |
| Stigmasterol | CYP27B1 | BATMAN-TCM |
| Stigmasterol | GC | BATMAN-TCM |
| Stigmasterol | SNW1 | BATMAN-TCM |
| Stigmasterol | AR | BATMAN-TCM |
| Stigmasterol | NR3C1 | BATMAN-TCM |
| Palmatine | NOS2 | TCMSP |
| Palmatine | PTGS1 | TCMSP |
| Palmatine | KCNH2 | TCMSP |
| Palmatine | ESR1 | TCMSP |
| Palmatine | AR | TCMSP |
| Palmatine | SCN5A | TCMSP |
| Palmatine | PTGS2 | TCMSP |
| Palmatine | NOS3 | TCMSP |
| Palmatine | RXRA | TCMSP |
| Palmatine | ADRB2 | TCMSP |
| Palmatine | ESR2 | TCMSP |
| Palmatine | HSP90AB1 | TCMSP |
| Palmatine | PRSS1 | TCMSP |
| Palmatine | PIM1 | TCMSP |
| Palmatine | NCOA2 | TCMSP |
| Palmatine | PRKACA | TCMSP |
| Palmatine | CDK2 | TCMSP |
| Palmatine | F7 | TCMSP |
| Palmatine | TH | HIT 2.0 |
| Palmatine | PDE10A | BATMAN-TCM |
| Palmatine | PDE4B | BATMAN-TCM |
| Palmatine | PDE4D | BATMAN-TCM |
| Quercetin | PTGS1 | TCMSP, HIT 2.0 |
| Quercetin | AR | TCMSP, HIT 2.0 |
| Quercetin | PPARG | TCMSP, HIT 2.0 |
| Quercetin | PTGS2 | TCMSP, HIT 2.0 |
| Quercetin | HSP90AB1 | TCMSP |
| Quercetin | PIK3CG | TCMSP, BATMAN-TCM |
| Quercetin | NCOA2 | TCMSP |
| Quercetin | DPP4 | TCMSP |
| Quercetin | AKR1B1 | TCMSP |
| Quercetin | PRSS1 | TCMSP |
| Quercetin | TOP2A | TCMSP, HIT 2.0 |
| Quercetin | F2 | TCMSP, HIT 2.0 |
| Quercetin | KCNH2 | TCMSP |
| Quercetin | SCN5A | TCMSP |
| Quercetin | F10 | TCMSP |
| Quercetin | ADRB2 | TCMSP |
| Quercetin | MMP3 | TCMSP |
| Quercetin | PRKACA | TCMSP |
| Quercetin | F7 | TCMSP |
| Quercetin | NOS3 | TCMSP, HIT 2.0 |
| Quercetin | RXRA | TCMSP |
| Quercetin | ACHE | TCMSP, HIT 2.0 |
| Quercetin | GABRA1 | TCMSP |
| Quercetin | MAOB | TCMSP |
| Quercetin | RELA | TCMSP, HIT 2.0 |
| Quercetin | EGFR | TCMSP, HIT 2.0 |
| Quercetin | AKT1 | TCMSP, HIT 2.0 |
| Quercetin | VEGFA | TCMSP, HIT 2.0 |
| Quercetin | CCND1 | TCMSP, HIT 2.0 |
| Quercetin | BCL2 | TCMSP, HIT 2.0 |
| Quercetin | BCL2L1 | TCMSP, HIT 2.0 |
| Quercetin | FOS | TCMSP, HIT 2.0 |
| Quercetin | CDKN3 | TCMSP |
| Quercetin | EIF6 | TCMSP |
| Quercetin | BAX | TCMSP, HIT 2.0 |
| Quercetin | CASP9 | TCMSP, HIT 2.0 |
| Quercetin | PLAU | TCMSP, HIT 2.0 |
| Quercetin | MMP2 | TCMSP, HIT 2.0 |
| Quercetin | MMP9 | TCMSP, HIT 2.0 |
| Quercetin | MAP3K7 | TCMSP |
| Quercetin | IL10 | TCMSP, HIT 2.0 |
| Quercetin | EGF | TCMSP, HIT 2.0 |
| Quercetin | RB1 | TCMSP, HIT 2.0 |
| Quercetin | TNF | TCMSP, HIT 2.0 |
| Quercetin | JUNB | TCMSP |
| Quercetin | IL6 | TCMSP, HIT 2.0 |
| Quercetin | CDKN2A | TCMSP, HIT 2.0 |
| Quercetin | AHSA1 | TCMSP |
| Quercetin | CASP3 | TCMSP, HIT 2.0 |
| Quercetin | TP53 | TCMSP, HIT 2.0 |
| Quercetin | ELK1 | TCMSP, HIT 2.0 |
| Quercetin | NFKBIA | TCMSP, HIT 2.0 |
| Quercetin | ODC1 | TCMSP, HIT 2.0 |
| Quercetin | XDH | TCMSP, HIT 2.0 |
| Quercetin | CASP8 | TCMSP, HIT 2.0 |
| Quercetin | TOP1 | TCMSP, HIT 2.0 |
| Quercetin | RAF1 | TCMSP, HIT 2.0 |
| Quercetin | SOD1 | TCMSP |
| Quercetin | PRKCA | TCMSP, HIT 2.0 |
| Quercetin | MMP1 | TCMSP, HIT 2.0 |
| Quercetin | HIF1A | TCMSP, HIT 2.0 |
| Quercetin | STAT1 | TCMSP, HIT 2.0 |
| Quercetin | RUNX1T1 | TCMSP |
| Quercetin | HERC5 | TCMSP |
| Quercetin | CDK1 | TCMSP, HIT 2.0 |
| Quercetin | HSPA5 | TCMSP, HIT 2.0 |
| Quercetin | ERBB2 | TCMSP, HIT 2.0 |
| Quercetin | ACACA | TCMSP |
| Quercetin | HMOX1 | TCMSP, HIT 2.0 |
| Quercetin | CYP3A4 | TCMSP, HIT 2.0 |
| Quercetin | CYP1A2 | TCMSP, HIT 2.0 |
| Quercetin | CAV1 | TCMSP, HIT 2.0 |
| Quercetin | MYC | TCMSP, HIT 2.0 |
| Quercetin | F3 | TCMSP, HIT 2.0 |
| Quercetin | GJC1 | TCMSP |
| Quercetin | CYP1A1 | TCMSP, HIT 2.0 |
| Quercetin | ICAM1 | TCMSP, HIT 2.0 |
| Quercetin | IL1B | TCMSP, HIT 2.0 |
| Quercetin | XCL2 | TCMSP |
| Quercetin | SELE | TCMSP, HIT 2.0 |
| Quercetin | VCAM1 | TCMSP, HIT 2.0 |
| Quercetin | PTGER3 | TCMSP |
| Quercetin | CXCL8 | TCMSP, HIT 2.0 |
| Quercetin | PRKCB | TCMSP, HIT 2.0 |
| Quercetin | BIRC5 | TCMSP |
| Quercetin | DUOXA2 | TCMSP |
| Quercetin | HSPB1 | TCMSP, HIT 2.0 |
| Quercetin | TGFB1 | TCMSP, HIT 2.0 |
| Quercetin | SULT1E1 | TCMSP, HIT 2.0 |
| Quercetin | MGAM | TCMSP |
| Quercetin | IL2 | TCMSP, HIT 2.0 |
| Quercetin | NR1I2 | TCMSP, HIT 2.0 |
| Quercetin | CYP1B1 | TCMSP, HIT 2.0 |
| Quercetin | CCNB1 | TCMSP, HIT 2.0 |
| Quercetin | PLAT | TCMSP, HIT 2.0 |
| Quercetin | THBD | TCMSP |
| Quercetin | SERPINE1 | TCMSP, HIT 2.0 |
| Quercetin | COL1A1 | TCMSP, HIT 2.0 |
| Quercetin | IFNG | TCMSP, HIT 2.0 |
| Quercetin | ALOX5 | TCMSP |
| Quercetin | PTEN | TCMSP, HIT 2.0 |
| Quercetin | IL1A | TCMSP, HIT 2.0 |
| Quercetin | MPO | TCMSP, HIT 2.0 |
| Quercetin | NCF1 | TCMSP, HIT 2.0 |
| Quercetin | ABCG2 | TCMSP, HIT 2.0 |
| Quercetin | HAS2 | TCMSP |
| Quercetin | GSTP1 | TCMSP, HIT 2.0 |
| Quercetin | NFE2L2 | TCMSP, HIT 2.0 |
| Quercetin | NQO1 | TCMSP, HIT 2.0 |
| Quercetin | PARP1 | TCMSP, HIT 2.0 |
| Quercetin | AHR | TCMSP, HIT 2.0 |
| Quercetin | PSMD3 | TCMSP |
| Quercetin | SLC2A4 | TCMSP, HIT 2.0 |
| Quercetin | COL3A1 | TCMSP |
| Quercetin | CXCL11 | TCMSP |
| Quercetin | CXCL2 | TCMSP, HIT 2.0 |
| Quercetin | DCAF5 | TCMSP |
| Quercetin | NR1I3 | TCMSP, HIT 2.0 |
| Quercetin | CHEK2 | TCMSP, HIT 2.0 |
| Quercetin | INSR | TCMSP, HIT 2.0 |
| Quercetin | CLDN4 | TCMSP, HIT 2.0 |
| Quercetin | PPARA | TCMSP, HIT 2.0 |
| Quercetin | PPARD | TCMSP |
| Quercetin | HSF1 | TCMSP, HIT 2.0 |
| Quercetin | CRP | TCMSP, HIT 2.0 |
| Quercetin | CXCL10 | TCMSP, HIT 2.0 |
| Quercetin | CHUK | TCMSP, HIT 2.0 |
| Quercetin | SPP1 | TCMSP, HIT 2.0 |
| Quercetin | RUNX2 | TCMSP, HIT 2.0 |
| Quercetin | RASSF1 | TCMSP, HIT 2.0 |
| Quercetin | E2F1 | TCMSP, HIT 2.0 |
| Quercetin | E2F2 | TCMSP, HIT 2.0 |
| Quercetin | ACP3 | TCMSP |
| Quercetin | CTSD | TCMSP, HIT 2.0 |
| Quercetin | IGFBP3 | TCMSP, HIT 2.0 |
| Quercetin | IGF2 | TCMSP |
| Quercetin | CD40LG | TCMSP, HIT 2.0 |
| Quercetin | IRF1 | TCMSP, HIT 2.0 |
| Quercetin | ERBB3 | TCMSP, HIT 2.0 |
| Quercetin | PON1 | TCMSP, HIT 2.0 |
| Quercetin | DIO1 | TCMSP |
| Quercetin | PCOLCE | TCMSP |
| Quercetin | NPEPPS | TCMSP, HIT 2.0 |
| Quercetin | HK2 | TCMSP, HIT 2.0 |
| Quercetin | NKX3-1 | TCMSP, HIT 2.0 |
| Quercetin | RASA1 | TCMSP |
| Quercetin | GSTM1 | TCMSP, HIT 2.0 |
| Quercetin | GSTM2 | TCMSP, HIT 2.0 |
| Quercetin | CYP2C8 | HIT 2.0 |
| Quercetin | MAPK1 | HIT 2.0 |
| Quercetin | PRKAA2 | HIT 2.0 |
| Quercetin | KDM1A | HIT 2.0 |
| Quercetin | MAP3K5 | HIT 2.0 |
| Quercetin | TXNIP | HIT 2.0 |
| Quercetin | SLC23A1 | HIT 2.0 |
| Quercetin | HSP90B2P | HIT 2.0 |
| Quercetin | HSP90AA1 | HIT 2.0 |
| Quercetin | TYR | HIT 2.0 |
| Quercetin | HSPA1A | HIT 2.0 |
| Quercetin | HSPA1B | HIT 2.0 |
| Quercetin | HSPA4 | HIT 2.0 |
| Quercetin | GK | HIT 2.0 |
| Quercetin | VDR | HIT 2.0 |
| Quercetin | PRKAA1 | HIT 2.0 |
| Quercetin | MTOR | HIT 2.0 |
| Quercetin | PRKCD | HIT 2.0 |
| Quercetin | JAK2 | HIT 2.0 |
| Quercetin | CRTC1 | HIT 2.0 |
| Quercetin | PGP | HIT 2.0 |
| Quercetin | AURKB | HIT 2.0 |
| Quercetin | SLC2A1 | HIT 2.0 |
| Quercetin | SLPI | HIT 2.0 |
| Quercetin | ERN2 | HIT 2.0 |
| Quercetin | CDH1 | HIT 2.0 |
| Quercetin | GAA | HIT 2.0 |
| Quercetin | CYP2C19 | HIT 2.0 |
| Quercetin | UGT1A9 | HIT 2.0 |
| Quercetin | SIRT1 | HIT 2.0 |
| Quercetin | LYN | HIT 2.0 |
| Quercetin | MAOA | HIT 2.0 |
| Quercetin | HVCN1 | HIT 2.0 |
| Quercetin | PTPN11 | HIT 2.0 |
| Quercetin | CYP2E1 | HIT 2.0 |
| Quercetin | LDLR | HIT 2.0 |
| Quercetin | COMT | HIT 2.0 |
| Quercetin | NRF1 | HIT 2.0 |
| Quercetin | CTNNB1 | HIT 2.0 |
| Quercetin | NFKB1 | HIT 2.0 |
| Quercetin | JUN | HIT 2.0 |
| Quercetin | NOS2 | HIT 2.0 |
| Quercetin | CDKN1A | HIT 2.0 |
| Quercetin | CAT | HIT 2.0 |
| Quercetin | CCL2 | HIT 2.0 |
| Quercetin | GSK3B | HIT 2.0 |
| Quercetin | POR | HIT 2.0 |
| Quercetin | MCL1 | HIT 2.0 |
| Quercetin | VIM2P | HIT 2.0 |
| Quercetin | CEBPB | HIT 2.0 |
| Quercetin | NLRP3 | HIT 2.0 |
| Quercetin | DNAJC5 | HIT 2.0 |
| Quercetin | HNRNPA1 | HIT 2.0 |
| Quercetin | MAPK3 | HIT 2.0 |
| Quercetin | FOSL1 | HIT 2.0 |
| Quercetin | MUC5AC | HIT 2.0 |
| Quercetin | MAPK14 | HIT 2.0 |
| Quercetin | USP10 | HIT 2.0 |
| Quercetin | MAPK8 | HIT 2.0 |
| Quercetin | PTK2B | HIT 2.0 |
| Quercetin | BMP2 | HIT 2.0 |
| Quercetin | TXN | HIT 2.0 |
| Quercetin | VIM | HIT 2.0 |
| Quercetin | XBP1 | HIT 2.0 |
| Quercetin | THBS1 | HIT 2.0 |
| Quercetin | ABCA1 | HIT 2.0 |
| Quercetin | STAT3 | HIT 2.0 |
| Quercetin | CRH | HIT 2.0 |
| Quercetin | BRCA1 | HIT 2.0 |
| Quercetin | SLC16A1 | HIT 2.0 |
| Quercetin | TNFRSF10B | HIT 2.0 |
| Quercetin | MLXIPL | HIT 2.0 |
| Quercetin | BDNF | HIT 2.0 |
| Quercetin | NR1H3 | HIT 2.0 |
| Quercetin | PTX3 | HIT 2.0 |
| Quercetin | ADIPOQ | HIT 2.0 |
| Quercetin | MECP2 | HIT 2.0 |
| Quercetin | UQCRC1 | HIT 2.0 |
| Quercetin | NDUFV2 | HIT 2.0 |
| Quercetin | TJP1 | HIT 2.0 |
| Quercetin | GABRR3 | HIT 2.0 |
| Quercetin | EIF2S1 | HIT 2.0 |
| Quercetin | P2RX4 | HIT 2.0 |
| Quercetin | APOE | HIT 2.0 |
| Quercetin | KHK | HIT 2.0 |
| Quercetin | GCK | HIT 2.0 |
| Quercetin | FASN | HIT 2.0 |
| Quercetin | SCD | HIT 2.0 |
| Quercetin | CREB1 | HIT 2.0 |
| Quercetin | EGR1 | HIT 2.0 |
| Quercetin | CLDN5 | HIT 2.0 |
| Quercetin | LEF1 | HIT 2.0 |
| Quercetin | AXIN1 | HIT 2.0 |
| Quercetin | AXIN2 | HIT 2.0 |
| Quercetin | IL17A | HIT 2.0 |
| Quercetin | ATF4 | HIT 2.0 |
| Quercetin | PIK3CB | HIT 2.0 |
| Quercetin | NGF | HIT 2.0 |
| Quercetin | ATP5B | BATMAN-TCM |
| Quercetin | STK17B | BATMAN-TCM |
| Quercetin | ATP5C1 | BATMAN-TCM |
| Quercetin | HIBCH | BATMAN-TCM |
| Quercetin | UGT3A1 | BATMAN-TCM |
| Quercetin | PIM1 | BATMAN-TCM |
| Quercetin | ATP5A1 | BATMAN-TCM |
| Quercetin | HCK | BATMAN-TCM |
